# mSphere of Influence: Turning to Soil for Medicines

**DOI:** 10.1128/mSphere.01258-20

**Published:** 2021-01-06

**Authors:** Ching-Hsuan Lin

**Affiliations:** a Department of Biochemical Science and Technology, College of Life Science, National Taiwan University, Taipei, Taiwan

**Keywords:** uncultivable microorganisms, iChip, new antibiotic, antimicrobial compounds

## Abstract

Ching-Hsuan Lin works in the field of *Candida* biology. In this mSphere of Influence article, he reflects on how the papers “Use of ichip for high-throughput *in situ* cultivation of uncultivable microbial species” by D. Nichols, N. Cahoon, E. M. Trakhtenberg, L. Pham, et al. (Appl Environ Microbiol 76:2445–2450, 2010, https://doi.org/10.1128/AEM.01754-09) and “A new antibiotic kills pathogens without detectable resistance” by L. L. Ling, T. Schneider, A. J. Peoples, A. L. Spoering, et al. (Nature 517:455–459, 2015, https://doi.org/10.1038/nature14098) made an impact on him by inspiring him to explore new bioactive antimicrobial compounds with his collaborators.

## Abstract (precis)

Ching-Hsuan Lin works in the field of *Candida* biology. In this mSphere of Influence article, he reflects on how the papers “Use of ichip for high-throughput *in situ* cultivation of uncultivable microbial species” by D.

## COMMENTARY

Although more than 5 × 10^30^ microorganisms have been predicted in our living place, only a few thousand can be cultivated in laboratories (1). Lewis and Epstein’s team designed a tiny diffusion chamber (Isolation Chip [iChip]) under conditions with or without specific growth factors to isolate uncultivated microorganisms (2). A new species, Eleftheria terrae, was thus successfully cultivated by using iChip. With this breakthrough, a new antibiotic compound, teixobactin, produced by *E. terrae*, was obtained from the extracts of 10,000 isolates, and the potential biosynthetic gene cluster of teixobactin was also identified after the genome of *E. terrae* was obtained (3). Lewis’s group further demonstrated *in vitro* and *in vivo* the antimicrobial mechanism of teixobactin, which involved inhibiting the synthesis of lipid II (precursor of peptidoglycan) and lipid III (precursor of teichoic acid) to effectively kill Gram-positive bacteria and their drug-resistant strains (3). Moreover, no teixobactin-resistant strain was obtained during *in vitro* passage, suggesting that teixobactin might reduce the likelihood that resistance will develop in the future (3). Two papers have significantly influenced my way of thinking and fueled my interest to pursue new possibilities in drug discovery.

Microbial metabolites are a rich and promising source for drug discovery. Most antibiotics we are using were discovered in 1940 to 1960, and hundreds of the most important clinical drugs are obtained from cultured microorganisms. Repeated screening for new antimicrobial drugs through the same methods and platforms within the same microorganisms resulted in catastrophic failure inevitably. Therefore, development of new techniques and platforms such as alternative culturing methods for a broader spectrum of microorganisms and the access to potential compounds via the expression of metagenomic libraries is a paramount task for the field (4). Hence, what was striking to me is that iChip is a novel, manipulatable, and effective platform for extensive isolation of uncultivable microorganisms. Indeed, the microbial growth recovery by iChip was 50 times higher than that in a standard petri dish (3). Teixobactin might not have been found without iChip. There is no doubt that together with collaborators, Lewis’s group opened a door to a new world for scientists who are enthusiastic and passionate about drug discovery.

Fungal infections have dramatically increased over the past few decades (5). Compared to the antibiotics in current use, antifungal drugs are limited, and the emergence of resistant strains is occurring worldwide. Development and discovery of novel antifungal agents are therefore an unmet need. Extracts of these bacterial isolates acquired from the iChip will be useful to discover new antifungal drugs, too. Not only that, the greatest thing is that the developed technique can be broadly applied to find new drugs for treatment of viral infections, cancers, and other diseases.

Starting a career in research as a Principal Investigator, my work was focusing on the *Candida* species to understand the molecular aspects and related signaling pathways in its sexual cycle and pathogenesis. The challenge I faced was that there always has been a gap between basic science (benchside) and clinical research (bedside). For example, drug development is a time-consuming, costly, and complex process. To translate fundamental scientific discoveries to clinical impacts, a multidisciplinary team is key to success. The breakthrough iChip technique certainly inspires scientists in academia like me to devote themselves to the discovery and development of new antimicrobial agents again. It certainly has impacted my career, leading me to change the research focus of my academic career. Currently, more than half of my laboratory projects are for applied science in the discovery and identification of new antifungal compounds and new therapeutic methods for the treatment of fungal infections via multidisciplinary collaboration.
